# Food grain quality: Analysis of physical, biometric, and colorimetric properties to promote consumption

**DOI:** 10.1016/j.heliyon.2024.e29234

**Published:** 2024-04-06

**Authors:** Nicodemo C. Jamanca-Gonzales, Robert W. Ocrospoma-Dueñas, Yolanda M. Eguilas-Caushi, Rossy A. Padilla-Fabian, Reynaldo J. Silva-Paz

**Affiliations:** Escuela de Ingeniería en Industrias Alimentarias, Departamento de Ingeniería, Universidad Nacional de Barranca, Av. Toribio de Luzuriaga N° 376 Mz J., Urb. La Florida, Barranca, Lima, Peru

**Keywords:** Seeds, Dendrogram, Biometry, Color, Morphology

## Abstract

This research focused on analyzing the biometric, colorimetric and morphological characteristics of thirty seeds, covering legumes, cereals and oilseeds. Thirteen legumes, fourteen cereals and three oilseeds were collected from three different locations. The methodology used was descriptive, applying multivariate multiple factorial and cluster analysis. The results showed variability between biometric, chromatic and morphological characteristics among the seeds. Predominant shapes include circular, oval, oblong, less frequently kidney and lanceolate. Significant differences in biometric parameters stand out, evidencing similarities in colorimetric parameters. Specifically, Pallar and Bean exhibited greater equatorial dimensions, length, weight, 100 g weight, area and perimeter, While peanut and Chickpea presented greater thickness. In terms of colorimetry - luminosity, Pallar, Yellow corn and Tarwi presented higher values unlike Black lentils, Purple corn and Black beans, being less luminous. Multivariate tests revealed the formation of four groups based on the parameters studied. This study provides valuable information about the different seeds, establishing a basis for their propagation and improvement in the Peruvian context.

## 1 Introduction

Seeds play an essential role in the reproduction of plants, they have nutrients (proteins, carbohydrates, vitamins, minerals and oils) that provide the energy and materials necessary for germination and subsequent development [1,2]. In the context of biodiversity, Peru stands out as a megadiverse country, hosting a rich variety of flora and fauna species. At a global level, there is a growing trend towards the preservation and protection of seed diversity, recognized as the fundamental source of food. The promotion of resilient food systems is premised on considering seeds as the life insurance of our food production, as highlighted by the Food and Agriculture Organization of the United Nations [3]. This approach reflects the critical importance of preserving seed biodiversity in the global context and highlights the vital interconnection between food security and the conservation of this invaluable gene pool.

To classify seeds there are criteria such as their origin (Angiosperms and Gymnosperms), their reserve substance situation (Endosperm, Exendosperm, Perisperm), number of cotyledons (monocotyledons, or dicotyledons) or according to their fruits (grains, pseudocereals or legumes), the latter being the one that shows interest for the food industry [4]. Seed quality is an agronomic concept linked to a set of physical, physiological, genetic and health attributes. Physical quality represents the appearance of the seed, which depends on size, volumetric weight, brightness, analytical purity, absence of seeds of common and harmful weeds, and other crops [5]. For their use in the food industry, they are called “grains”, their characterization being very necessary, as it allows determining their aptitudes for industrial processing.

The main grains for food and industrial use are cereals (grass family), pseudocereals, legumes and oilseed grains. Cereals par excellence constitute an important source of carbohydrates and are highly recommended for their nutritional and health benefits for the diet [6]. Pseudocereals known as cereals or Andean grains are an important source of macronutrients, vitamins, minerals and mainly proteins [7]. Legumes constitute an important source of protein, with tarwi and soybeans considered high protein due to their protein content above 40% [8], oilseed grains are also important sources of fat, being used industrially for the production of oils.

Morphological characterization allows determining their quality, likewise the size of the grains, specified by the weight of 100 grains, allows grains to be classified by quality, so for example in the case of beans they are classified into 3 groups: small (up to 25 g/100 seeds), medium (between 25 and 40 g/100 seeds) and large (from 40 g/100 seeds) [9]. These characteristics in the agronomic aspect influence their germination capacity [10], while in industrial processes they directly influence grinding, cooking, nutrition and appearance [11]. The shape and size descriptors of the seeds can be analyzed by image analysis using statistical techniques such as descriptive statistics, principal components, Euclidean distance, Mantel correlation test and supervised machine learning, with the image analysis technique being effective for detect biometric differences between seeds [12]. Likewise, computer vision techniques are being used to evaluate the morphometric and colorimetric characteristics of seeds that describe the shape, size and textural features of the seeds [13], and there are also studies on morpho characterizations, colorimetric measurements of biological species [14], in addition the images are being used to analyze the texture, morphology and color of the vein, there are studies that use images of the seed, considering attributes such as perimeter, area, diameter and centroid, are used to analyze the quality of the seeds using machine learning [15].

The color of the grains is very varied, with shades of white, cream, yellow, brown, pink, red, purple, black observed in legumes due to the presence of phytochemicals [9]. In relation to cereals such as quinoa, in Peru there is great biological diversity, with a wide variety of colors such as: yellow, cream, black, reddish, translucent or white [16], with data from CIELab color of white (L = 80.002; a = −0.752; b = 11.517), red (L = 48.727; a = 30.487; b = 41.191), black quinoa (L = 20.515; a = −0.974; b = −2499) [17], while for a good group of seeds there are few reports of their color, for whose quantification in some cases the Methuen Handbook of Color chart has been used [16,18], based on a subjective color assessment, guided by common names, however, there is limited literature on the chromatic characterization of grains for food use.

Considering the industrial importance of food grains for the processing of various intermediate products such as flour, and these in turn for various processed products, the objective of this work was to evaluate the biometric and colorimetric characteristics by images of thirty (30) grains of food consumption, applying descriptive statistical tools and principal components.

## 2 Materials and methods

### 2.1 Sample

This study involved the evaluation of thirty (30) grain seeds, of which thirteen (13) were legumes, fourteen (14) were cereals and three (03) were oilseed grains, described in Table 1. The seeds were collected from three centers for the sale and marketing of food products: Huaraz, Barranca and Lima, Peru, between the months of April and May 2023. The grain production occurs along the inter-Andean and coastal valleys of Peru. The collection was carried out by the authors and students of the Professional School of Engineering in Food Industries of the National University of Barranca, Peru. The seeds were naturally dried with a water content of around 8–10% [1] during the collection period [19] and stored under ambient conditions until the measurements were carried out.

**Table 1:**
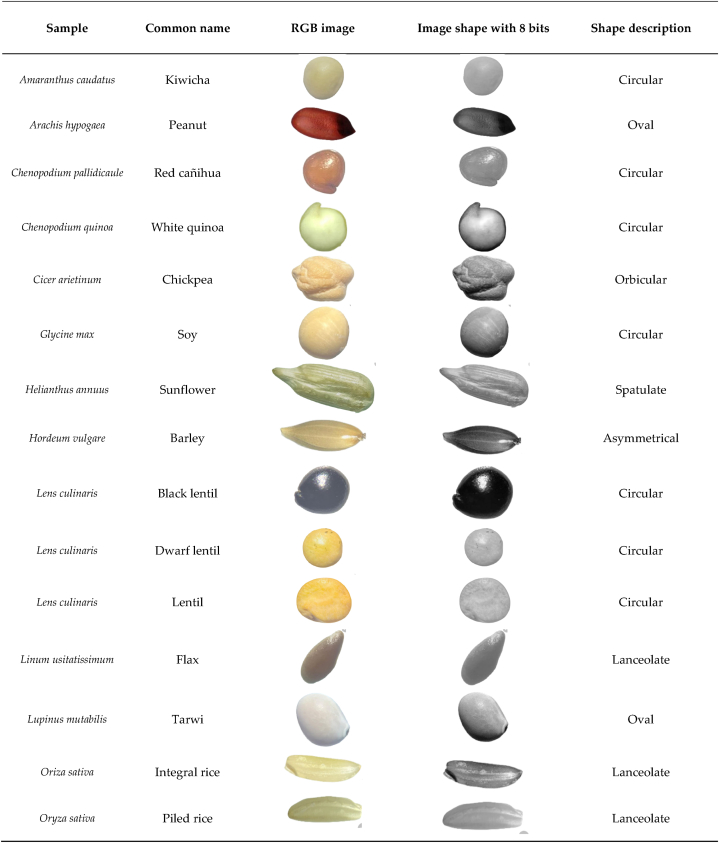

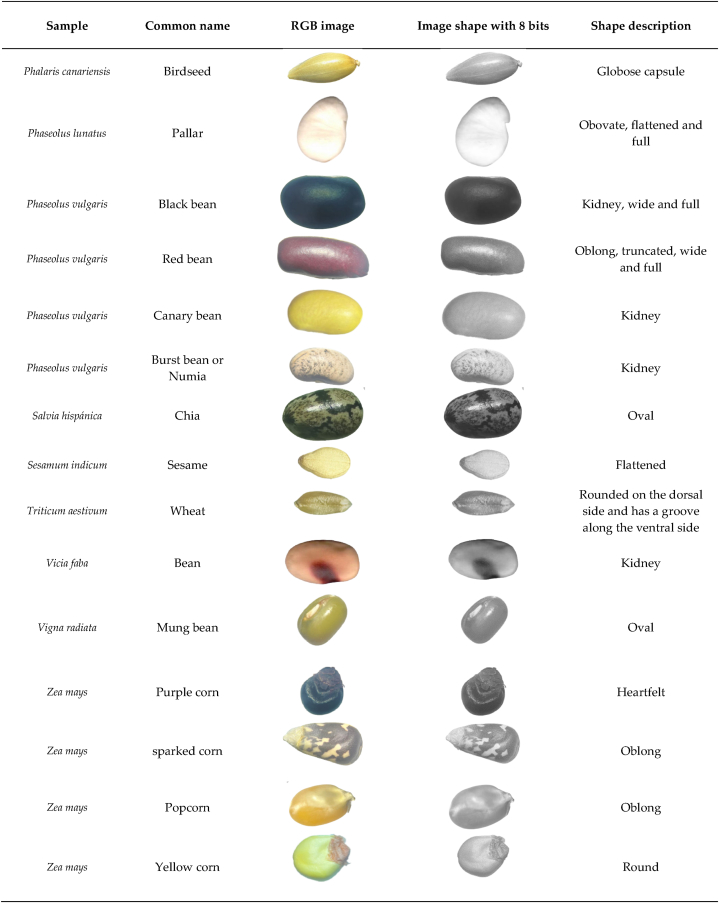
Physical structure and morphological description of the grains (Images seen with a magnification of 25×).

### 2.2 Biometric grain analysis

The biometric measurements of major or equatorial diameter (mm), and minor diameter were measured using a previously calibrated micrometer (Electronic Outside Micrometer, sensitivity 0.001 mm, model 140706991, Peru), with a scale of 0–25 mm. The weight and biomass or one hundred seeds were weighed individually per grain using an analytical balance with a sensitivity of 0.0001 g (Sartorius brand, model Entris 224-1S, Germany), according to the methodology reported by Morales-Santos et al. [20] and Pinzón et al. [21].

### 2.3 Image analysis

Images of small grains were determined using a Stemi DV4 Stereomicroscope (Brand Carl Zeiss Microscopy GmbH, Germany) with a zoom of 8X – 32X, while larger grains were taken with Scaner Laser Jet Pro (Model: 400 MFP, Series M425 DN, China), using a black background cover (0.25 m × 0.30 m), natural lighting and a 300x zoom. Shape and area analysis were performed from the images; the brightness, contrast and threshold of the images were adjusted and converted to 8-bit binary images to be analyzed using the particle analysis tool of ImageJ Software. ® (https://imagej.nih.gov/ij/). The area of the grains was calculated as the total area of the image [1,22]. The characteristics of the grains were evaluated according to what was described by Ubiergo & Lapp [23], contributions by Ferreira et al. [24], and Pablo-Pérez et al. [25], in addition, the terms to describe the shapes have been taken as reference from the descriptions used by Hoseney [26], Scade [27] and Kent [28]. The Remove BG online software was used to cut out the shapes, available at the following link https://pixlr.com/es/remove-background/.

### 2.4 Colorimetric analysis

The color parameters of the grains were quantified with the PCE Instruments Colorimeter (Model CSM 3, Spain) observation angle of 8°, blue LED D65 illuminant with 8 mm aperture. The color coordinates L* (0 = black, 100 = white), a* (+red, –green) and b* (+yellow, –blue) were determined according to the CIELAB coordinate color space system [29,30]. Additionally, the C*, h* was determined, where C* is the chroma and h* denotes the hue or angle of a polar measurement. The chroma value C* = (a*^2^ + b*^2^)^0.5^ and the hue angle h* = arctangent (b*/a*) [31]. The simulated color was determined using the Nixsensor online application available at the following link https://www.nixsensor.com/free-color-converter/and measured in quintuplicate at room temperature.

### 2.5 Statistical analysis

A statistical approach was used to discern differences between samples, opting for a completely randomized design with a 95% confidence level. In cases of statistically significant differences, a multiple comparison of means was carried out using the Tukey test. In addition, a multivariate multiple factorial analysis was applied, and to identify grouping patterns, a dendrogram was implemented. These analyzes were carried out on cereal samples [32], as well as on native potatoes [33], using cluster analysis that incorporates branching and a pie chart based on the principal component analysis methodology. Additionally, a heat map was used to visualize the relationships between the various grains. All statistical evaluations were executed using the XLSTAT 2023 program and Rproject with RStudio, ensuring accurate and detailed analysis of the data sets.

## 3 Results and discussion

### 3.1 Features of grain images

Grains such as cereals, pseudocereals, legumes and oilseeds show different characteristics among the studied: shapes, sizes, color intensity, and mainly their morphology. This behavior was described by Fernández et al. [32] in corn lines and Khatun et al. [34] in beans. Although the shape and size of the grains may be different, all cereal and legume grains of the same species have a similar structure and nutritional value [35]. The shapes described vary from a very regular, almost spherical shape that corresponds to lentils or soybeans, to more irregular shapes such as chickpeas. The analysis of seed morphology is a very important and conservative character [36,37].

### 3.2 Biometric parameters of grains

Table 2 presents the data obtained from the biometric characteristics of 30 grains for food use, considering 5 characteristics: largest or equatorial diameter, length, thickness, unit weight, weight of 100 grains, grain area and perimeter. These characteristics are essential to estimate processing yields and are closely related to their physical properties. Larger and heavier seeds usually store a greater amount of nutritional material, which results in increased yield during processing [38]. In soybeans or sunflowers, the oil content in the seeds exerts a significant influence on the extraction and yield of the process [39]. Likewise, in grains such as corn, both the quality and quantity of endosperm can influence the quantity of final products obtained during processing [40]. For legumes such as beans, the amount of protein present in the seeds can affect both the texture and yield of end products such as bean puree or protein isolate [41]. These interrelationships between seed characteristics and processing outcomes are critical to understanding and optimizing agricultural and food processes. Significant differences are observed in all the parameters studied. Among legume grains, Pallar displays the highest measurements compared to other varieties. Similarly, within the cereal group, purple corn exhibits superior results, while peanuts stand out among oilseed grains. This same behavior of maximum values occurs for the other characteristics. This heterogeneity in grains occurs mainly due to physiological, genetic and environmental factors [42].

**Table 2:** Biometric characteristics of the different grains studied.

Sample	Major or equatorial diameter (mm)	Length (mm)	Thickness (m)	Weight (g)	Weight of 100 grains (g)	Grain area (mm^2^)	Perimeter (mm)
Kiwicha	1.49 ± 0.19^lmn^	1.01 ± 0.14^p^	1.01 ± 0.14^jk^	0.01 ± 0.03^j^	0.10 ± 0.03^j^	1.47 ± 0.21^n^	4.36 ± 0.34^q^
Peanut	10.48 ± 1.08^c^	15.47 ± 1.59^b^	9.58 ± 1.15^a^	0.93 ± 0.20^b^	92.63 ± 20.04^b^	113.78 ± 14.27^de^	46.89 ± 4.11^b^
Red cañihua	0.72 ± 1.33^n^	0.72 ± 0.06^p^	0.72 ± 0.06^k^	0.01 ± 0.01^j^	0.06 ± 0.01^j^	1.46 ± 0.24^n^	4.33 ± 0.44^q^
White quinoa	2.46 ± 0.23^kl^	1.26 ± 0.11^p^	1.26 ± 0.11^jk^	0.01 ± 0.01^j^	0.49 ± 0.08^j^	4.10 ± 0.74^mn^	7.49 ± 0.77^o^
Chickpea	9.44 ± 0.55^d^	12.26 ± 0.78^cd^	9.37 ± 0.49^a^	0.75 ± 0.12^c^	74.98 ± 11.54^c^	82.16 ± 10.95^f^	37.49 ± 3.43^e^
Soy	6.72 ± 0.34^f^	6.72 ± 0.34^ij^	5.42 ± 0.18^e^	0.12 ± 0.02^hij^	12.43 ± 2.39^hij^	29.09 ± 2.56^j^	20.18 ± 0.90^j^
Sunflower	4.88 ± 0.38^gh^	10.71 ± 0.84^de^	2.34 ± 0.21^ghi^	0.07 ± 0.01^ij^	6.69 ± 1.23^ij^	38.57 ± 6.55^i^	27.37 ± 2.13^g^
Barley	3.01 ± 0.20^jk^	10.93 ± 0.71^de^	3.01 ± 0.20^fg^	0.05 ± 0.01^ij^	5.33 ± 0.47^ij^	29.79 ± 4.67^j^	27.45 ± 2.84^g^
Black lentil	3.53 ± 0.24^ij^	3.53 ± 0.24^mno^	2.26 ± 0.13^ghi^	0.02 ± 0.01^ij^	2.15 ± 0.27^ij^	17.85 ± 2.49^k^	15.98 ± 1.19^l^
Dwarf lentil	4.29 ± 0.31^hi^	4.29 ± 0.31^lmn^	2.17 ± 0.16^ghi^	0.03 ± 0.01^ij^	2.89 ± 0.51^ij^	15.63 ± 1.76^k^	14.73 ± 0.91^lm^
Lentil	6.31 ± 0.36^f^	6.31 ± 0.36^jk^	2.35 ± 0.13^gh^	0.07 ± 0.01^ij^	7.04 ± 1.06^ij^	33.29 ± 4.26^ij^	22.02 ± 1.69^i^
Flax	2.45 ± 0.21^kl^	5.03 ± 0.33^klm^	1.02 ± 0.15^jk^	0.01 ± 0.01^j^	0.65 ± 0.12^j^	7.94 ± 1.21^lm^	12.10 ± 0.95^n^
Tarwi	7.89 ± 0.39^e^	10.03 ± 0.38^efg^	5.56 ± 0.49^de^	0.30 ± 0.04^fg^	30.29 ± 3.88^fg^	61.31 ± 7.59^g^	30.12 ± 1.76^f^
Integral rice	1.85 ± 0.05^lm^	8.06 ± 0.63^hi^	1.85 ± 0.05^hij^	0.02 ± 0.01^ij^	2.41 ± 0.15^ij^	15.17 ± 0.99^k^	18.34 ± 0.76^k^
Piled rice	2.39 ± 0.36^kl^	8.16 ± 0.55^hi^	1.77 ± 0.05^hij^	0.02 ± 0.01^ij^	2.26 ± 0.09^ij^	14.25 ± 1.12^k^	18.14 ± 1.27^k^
Birdseed	2.37 ± 0.19^kl^	5.59 ± 0.29^jkl^	1.49 ± 0.04^ijk^	0.01 ± 0.01^j^	0.77 ± 0.05^j^	7.56 ± 0.84^lm^	12.25 ± 0.80^n^
Pallar	16.26 ± 1.38^a^	23.33 ± 2.32^a^	6.39 ± 0.29^cd^	1.59 ± 0.32^a^	159.50 ± 32.40^a^	268.30 ± 56.40^a^	63.13 ± 7.39^a^
Black bean	6.50 ± 0.46^f^	10.13 ± 0.85^ef^	4.95 ± 0.38^e^	0.25 ± 0.04^fgh^	24.53 ± 3.50^fgh^	49.01 ± 5.31^h^	27.12 ± 1.51^g^
Red bean	8.01 ± 0.80^e^	15.22 ± 2.08^b^	6.56 ± 1.04^c^	0.67 ± 0.12^cd^	67.06 ± 12.30^cd^	121.36 ± 21.58^cd^	44.08 ± 4.19^c^
Canary bean	7.97 ± 0.38^e^	12.74 ± 1.03^c^	6.73 ± 0.33^c^	0.55 ± 0.07^d^	54.68 ± 7.29^d^	85.82 ± 8.22^f^	36.66 ± 1.70^e^
Burst bean or Numia	9.19 ± 0.68^d^	14.98 ± 1.04^b^	8.29 ± 0.63^b^	0.91 ± 0.16^b^	90.79 ± 15.70^b^	77.26 ± 11.97^f^	41.25 ± 5.44^d^
Chia	1.21 ± 0.16^mn^	2.21 ± 0.18^op^	0.78 ± 0.04^k^	0.01 ± 0.01^j^	0.1040 ± 0.0195^j^	2.02 ± 0.31^n^	5.74 ± 0.76^p^
Sesame	1.81 ± 0.22^lm^	5.59 ± 0.29^jkl^	0.83 ± 0.09^k^	0.01 ± 0.01^j^	0.2635 ± 0.0423^j^	1.79 ± 0.22^n^	5.16 ± 0.31^pq^
Wheat	3.33 ± 0.49^ijk^	8.38 ± 0.55^gh^	3.33 ± 0.49^f^	0.05 ± 0.01^ij^	5.3360 ± 0.9270^ij^	18.11 ± 2.46^k^	19.21 ± 1.33^jk^
Bean	13.91 ± 0.88^b^	22.2710 ± 1.94^a^	8.03 ± 0.89^b^	1.55 ± 0.18^a^	154.99 ± 18.01^a^	250.40 ± 38.46^b^	65.46 ± 8.83^a^
Mung bean	3.74 ± 0.27^ij^	4.71 ± 0.29^klmn^	3.71 ± 0.21^f^	0.05 ± 0.01^ij^	4.76 ± 0.62^ij^	12.44 ± 1.36^kl^	13.42 ± 0.80^mn^
Purple corn	9.66 ± 0.34^cd^	11.12 ± 0.68^cde^	4.92 ± 0.26^e^	0.27 ± 0.02^fgh^	26.56 ± 2.15^fgh^	83.19 ± 7.42^f^	35.73 ± 2.43^e^
Sparked corn	8.80 ± 1.23^de^	15.78 ± 1.85^b^	6.55 ± 1.18^c^	0.37 ± 0.07^ef^	37.28 ± 6.53^ef^	104.73 ± 21.55^e^	48.07 ± 6.67^b^
Popcorn	5.76 ± 0.46^fg^	8.73 ± 0.48^fgh^	5.01 ± 0.54^e^	0.18 ± 0.02^ghi^	17.74 ± 2.25^ghi^	37.59 ± 4.70^i^	24.31 ± 1.84^h^
Yellow corn	9.22 ± 1.20^d^	15.92 ± 1.53^b^	6.84 ± 1.03^c^	0.52 ± 0.14^de^	51.80 ± 51.80^de^	126.94 ± 23.68^c^	47.38 ± 4.35^b^

Valor = media ±SE, n = 50, Different letters in the same column indicate significant differences.

### 3.3 Colorimetric parameters of the grains

Grain color is related to the presence of phenolic components in the seed coat [43,44], shown visually as pigments, such as anthocyanins in soybean [45], in beans due to the presence of glycosides, flavonoids, anthocyanins and condensed tannins [44], while yellow beans contain carotenoids, red and black beans have anthocyanins, while white beans lack of these pigments [46,47]. The color of vetch faba is closely linked to the type of variety, with a direct relationship to the edaphoclimatic conditions of the production areas, thus the color (L*: Luminosity, a*: red/green coordinates, and b*: yellow/blue coordinates) is very close to that reported by Álvarez-Sánchez et al. [48].

Table 3 shows the color parameters in the CIELAB scale of the international system, both L*, a*, b*, C* and h* at the surface level of the pericarp (shell), a variability is evident in the luminosity of the grains and the parameters, a, b, C and h. Color variability depends on the pigment in the seed coat, its presence and type being a genetic function of the producer [26].

**Table 3:**
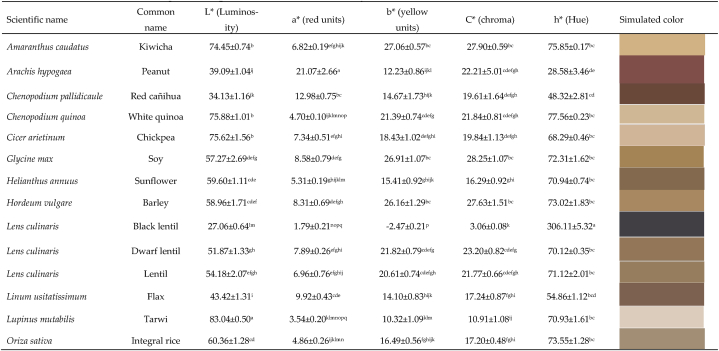

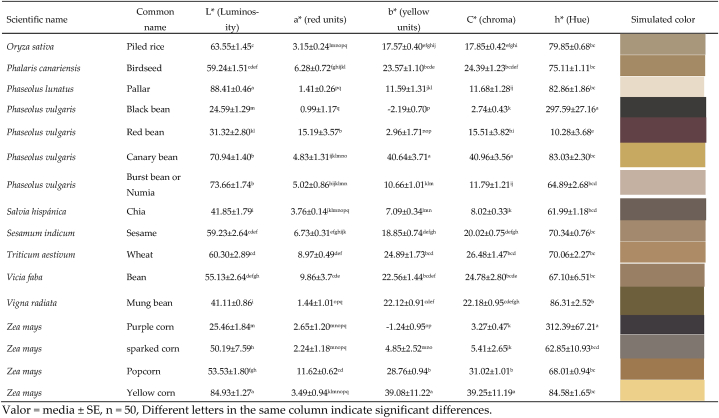
Color of the rind of grains expressed in the CIELab* system.

All colorimetric parameters presented significant differences. Of the 30 seeds, the lightest colors with high luminosity (L*>80) were Pallar, Yellow corn and Tarwi; while the darkest samples (L*<30) were black lentil, black bean and purple corn. The samples of Peanuts, Red beans, Red cañihua and Popcorn were the ones that presented the highest shades of red (a*>10), while there were no grains with fixed shades of green (values -a*). In relation to the b* parameter, the canary bean and yellow corn samples presented the highest values of the yellow tone (b*>35), while no blue tones were evident (-b* values). The highest chroma values (C*>30) were identified in Canary beans and Popcorn, whose trend was similar to the b* parameter. The highest values of the hue angle h* (h*>295) were recorded in Black lentils, Black beans and Purple corn, whose relationship is direct with the darker samples (L*<30). The variation in the color tones of the grains is related to the content and distribution of pigments at the level of the covering or shell, anthocyanins, glycosides, flavonoids, tannins [44,49], which directly influence their appearance.

**Fig. 1:**
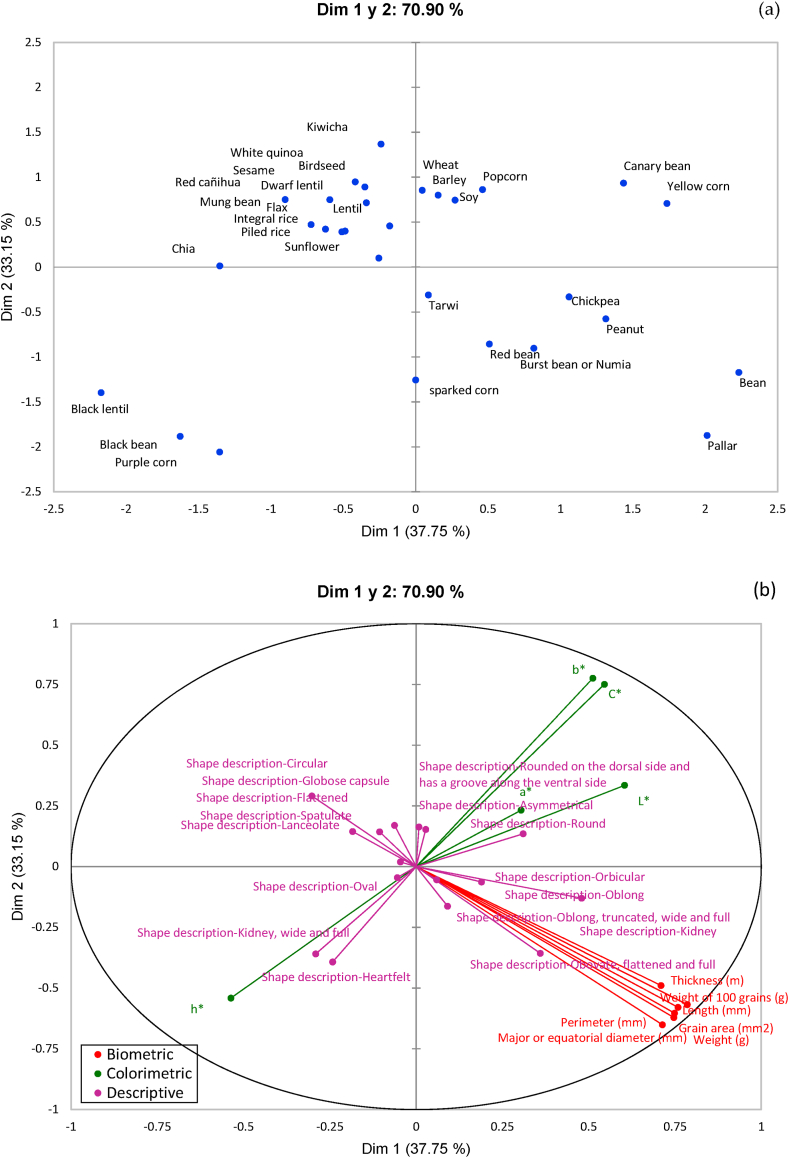
Multifactorial analysis of grains (a) and their shape, biometric and colorimetric descriptors (b).

To perform a correspondence analysis of the 13 biometric characteristics (major or equatorial diameter, length, thickness, weight, 100 grain weight, grain area and perimeter), colorimetric (L, a, b, C, h) and descriptive characteristics (shape) of the grains, multifactorial analysis and its descriptors were carried out. Fig. 1(a) illustrates the multifactorial analysis of grains by species, showing the projection of the observations in a two-dimensional space that explains 70.90% of the total variability of the data. Samples that are clustered close together in the graph share similar characteristics, while those that are more distant exhibit greater disparities. The formation of homogeneous groups is evident, highlighting the first group of small grains such as White quinoa, Canary seed, Red cañihua, Sesame, Dwarf lentil, Lentil, Sunflower and Kiwicha. The second group includes larger grains such as Pallar and Broad bean. The third group includes the Black lentil, Black bean and Purple corn samples. The fourth group is made up of Canary seed and Yellow corn. The fifth group includes the rest of the samples such as tarwi, peanut, Red bean, Sparked corn, Chickpea, Burst bean or Numia. Fig. 1(b) presents the groups according to shape, biometric and colorimetric descriptors, revealing variability in the shape descriptors, even within each group of grains (cereals, pseudocereals, legumes, oilseed grains). Five groups are identified: the first with circular, globose, flattened, spatula and lanceolate shapes; the second with obovate, flattened and complete shapes, with optimal biometric characteristics; the third group with oval, kidney-shaped and heart-shaped shapes, standing out with the h* parameter; the fourth group with asymmetrical and rounded shapes, predominating the parameters b*, C*, a* and L*; and finally, the fifth group characterized by orbicular, oblong, truncated and kidney shapes. The evaluated attributes influence the quality and appearance of the grains [50,51] and are what define its processing capacity.

**Fig. 2:**
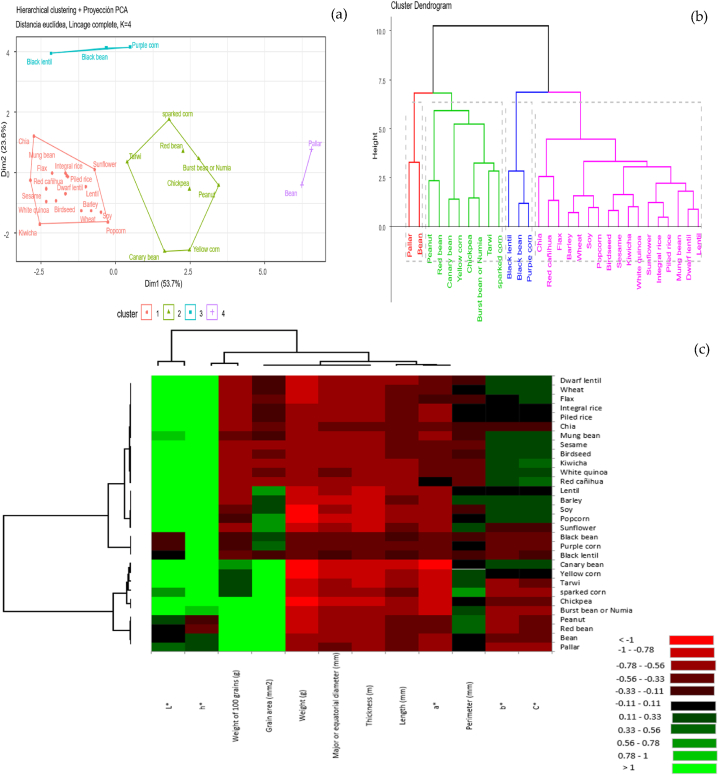
Analysis of ellipses using principal components (a), dendrogram applying cluster analysis (b) and heat map analysis (c) of the response variables applied to the different seeds.

Fig. 2 shows the multiple factorial multivariate analysis (a) and the dendrogram (b) obtained after clustering. Fig. 2(a) shows the formation of four groups: The first group made up of 17 grains (chia, red cañihua, linseed, barley, wheat, soy, popcorn, canary seed, sesame, kiwicha, white quinoa, sunflower, brown rice, ground rice, mung beans, dwarf lentils and lentils). The second group made up of 8 grains (peanuts, red beans, canary beans, yellow corn, chickpeas, numia, tarwi and sparkling corn), the third group made up of 2 grains (pallar and broad bean) and the fourth group made up of 3 grains (black lentil, black bean and purple corn). To carry out a more detailed analysis of the correspondences of all the biometric characteristics presented by the grains, conglomerate analysis was carried out using clustering. The dendrogram shows the groups that are formed by creating clusters of the biometric characteristics (diameter, length, thickness, weight and 100 grain weight) of the seeds and their similarity levels. The similarity level is measured on the vertical axis (alternatively the distance level can be displayed) and the different features are specified on the horizontal axis). Biometric characteristics are important both in the industrial field of processing to evaluate yields, as well as in the agronomic field as a “seed” to evaluate its germination capacity [52] and establishment of seedlings [53].

Fig. 2(b) shows a final partition of 4 clusters, which occurs at a similarity level of 80, which is higher than that reported by Gonzales & Peña [54] who obtained a dendrogram of genetic grouping of native potatoes using a similarity of 65, while Fernández et al. [32] obtained 5 clusters with 89 similarities, for the determination of the genetic relationship between corn lines using markers. The first conglomerate (far left) is made up of 2 grains (Pallar and Broad bean); the second conglomerate, in turn made up of 4 subconglomerates made up of 8 grains (Peanuts, Red beans, Canary beans, Yellow corn, Chickpeas, Numia, Tarwi and Sparkling corn); the third conglomerate made up of 3 grains (Black lentil, Black bean and Purple corn) and the fourth conglomerate made up of 10 subconglomerates, this being the most numerous of all, made up of 17 grains (Chia, Red cañihua, Linseed, Barley, Wheat, Soybeans, Popcorn, Canary seed, Sesame, Kiwicha, white quinoa, sunflower, brown rice, pilafed rice, mung beans, dwarf lentils and lentils). If the groups of the dendrogram were increased higher, then there would be fewer final clusters, but their level of similarity would be lower. If the dendrogram were reduced further down, then the level of similarity would be higher, but there would be more final clusters. Fig. 2(c) shows the heat map generated by the studied parameters of the 30 seeds. Color coding was graded on the red to green scale. The relative intensity increased from low (red) to high (green). The 30 seed samples were divided into four groups (left dendrogram) and the studied parameters were divided into three groups (upper dendrogram), the large group on the right corresponds to the colorimetric and biometric parameters. The perimeter shows intermediate values for most of the samples and higher values were for L* and h*, although these were lower for Pallar, Bean, Red bean, Peanut, Black bean, Black lentil, Purple corn and Mung bean. The advantage of using heat maps is that differences within the same group can be visualized quickly. For example, the parameters of 100 grain weight and total area are higher for Chickpea, Burst bean, Peanut, Red bean, Bean and Pallar, compared to the rest of the samples. These differences may be related to the different colorimetric parameters of the seeds. This technique has already been successfully applied in wine grouping to investigate the quality determinants of chenin blanc and pinotage wines [55] and in swedish beers [56], also in the evaluation of the quality of rice grains [11].

## 4 Conclusions

Grains constitute the vital reserve for the continuity of plant species, both cereals, legumes and oilseeds, which is why their biometric, colorimetric and shape characterization is important. The 30 grains studied were grouped into 13 legumes, 14 cereals and 3 oilseeds. These characteristics are essential for estimating processing performance, and they have a close relationship between all the characteristics. The images of the grains present great variability in terms of shape and intensity of the very heterogeneous coloration. The dendrogram analysis allowed four clusters to be obtained: the first cluster composed of 2 grains (Pallar and broad bean); the second conglomerate, in turn made up of 4 subconglomerates made up of 8 grains (Peanuts, Red beans, Canary beans, Yellow corn, Chickpeas, Numia, Tarwi and Sparkling corn); the third conglomerate made up of 3 grains (Black lentil, Black bean and Purple corn) and the fourth conglomerate made up of 10 subconglomerates, this being the most numerous of all, made up of 17 grains (Chia, Red cañihua, Linseed, Barley, Wheat, Soybeans, Popcorn, Canary seed, Sesame, Kiwicha, White quinoa, Sunflower, Brown rice, Pilafed rice, Mung beans, Dwarf lentils and Lentils).

## Funding

This article was developed thanks to the support provided by the Vice-Presidency of Research of the Universidad Nacional de Barranca through the financing of the Special Research Project "Formulation and elaboration of the functional panettone enriched with quinoa (*Chenopodium quinoa w*.) and amaranth flakes (*Amaranthus caudatus l.*)", thanks for the technical, academic and financial support.

## Informed consent statement

**N**ot applicable.

## Data availability statement

The data is contained in the article.

## Disclaimer/publisher's note

The statements, opinions and data contained in all publications are solely those of the individual author(s) and contributor(s) and not of MDPI and/or the editor(s). MDPI and/or the editor(s) disclaim responsibility for any injury to people or property resulting from any ideas, methods, instructions or products referred to in the content.

## CRediT authorship contribution statement

**Nicodemo C. Jamanca-Gonzales:** Writing – original draft, Investigation, Data curation, Conceptualization. **Robert W. Ocrospoma-Dueñas:** Resources, Investigation, Formal analysis, Data curation. **Yolanda M. Eguilas-Caushi:** Software. **Rossy A. Padilla-Fabian:** Visualization, Resources, Investigation, Conceptualizati. **Reynaldo J. Silva-Paz:** Writing – review & editing, Visualization, Project administration, Data curation, Conceptualization.

## Declaration of competing interest

The authors declare that they have no known competing financial interests or personal relationships that could have appeared to influence the work reported in this paper.
